# A metadata approach for clinical data management in translational genomics studies in breast cancer

**DOI:** 10.1186/1755-8794-2-66

**Published:** 2009-11-30

**Authors:** Irene Papatheodorou, Charles Crichton, Lorna Morris, Peter Maccallum, METABRIC Group, Jim Davies, James D Brenton, Carlos Caldas

**Affiliations:** 1 Department of Oncology, University of Cambridge and Cancer Research UK Cambridge Research Institute, Li Ka Shing Centre, Cambridge, CB2 0RE, UK; 2 Oxford University Computing Laboratory, Wolfson Building, Parks Road, Oxford, OX1 3QD, UK

## Abstract

**Background:**

In molecular profiling studies of cancer patients, experimental and clinical data are combined in order to understand the clinical heterogeneity of the disease: clinical information for each subject needs to be linked to tumour samples, macromolecules extracted, and experimental results. This may involve the integration of clinical data sets from several different sources: these data sets may employ different data definitions and some may be incomplete.

**Methods:**

In this work we employ semantic web techniques developed within the CancerGrid project, in particular the use of metadata elements and logic-based inference to annotate heterogeneous clinical information, integrate and query it.

**Results:**

We show how this integration can be achieved automatically, following the declaration of appropriate metadata elements for each clinical data set; we demonstrate the practicality of this approach through application to experimental results and clinical data from five hospitals in the UK and Canada, undertaken as part of the METABRIC project (Molecular Taxonomy of Breast Cancer International Consortium).

**Conclusion:**

We describe a metadata approach for managing similarities and differences in clinical datasets in a standardized way that uses Common Data Elements (CDEs). We apply and evaluate the approach by integrating the five different clinical datasets of METABRIC.

## Background

The METABRIC study (Molecular Taxonomy of Breast Cancer International Consortium) is an example of molecular profiling studies on cancer patients that aim to associate experimental results with clinical datasets in order to understand the clinical heterogeneity of the disease. The patient cohorts used are large and the clinical information is consolidated from a number of hospital databases that use different data definitions and often hold incomplete datasets. Patient information is often scattered in different databases within a hospital, or even between different hospitals, as patients are not necessarily treated by the same hospital throughout the course of their disease and/or relapse. Moreover, patient cohorts usually span a large period of time and depending on when the hospital started to record patient data electronically, this can result in incomplete clinical datasets. In addition to these, standard treatment and diagnosis procedures have changed throughout the last three decades, resulting in different types of information being accumulated over time. An example is the HER2 bio-marker assay, which has been a standard test, recorded at diagnosis, in our collaborating hospitals in Canada for the last few years, whereas only recently it has started being assessed regularly in the collaborating UK hospitals.

The different hospital databases have not been designed to conform to some agreed standards for data representation and meanings. They have been developed to fulfill the specific requirements of the hospital unit, and the data is described according to the definitions of assessments and concepts that are used locally. An example, is the use of Nottingham Prognostic Index (NPI) [1] versus the TNM classification scheme [2]. The databases we encountered in this study record either of those but never both. In addition to this, the TNM classification scheme is updated every few years, so any samples obtained at different times will have been classified by different TNM versions. The lack of common standards for cancer data representation and also the lack of a standardised means for relating from one classification scheme to another makes data sharing and integration a challenging task.

The semantic web is a term used to describe a collection of initiatives and technologies aimed at associating data with some representation of its meaning, so that we might access or process the data on the basis of its semantics, rather than its form or location. Although the technologies of the semantic web, such as the Resource Description Framework (RDF) [3,4] and the Web Ontology Language (OWL) [5] are still very much under development, they are already being applied widely in business, government, and scientific contexts [6].

Two Cancer Informatics projects, caBIG [7-10] and CancerGrid [11-13], are employing semantic web technology aiming to develop tools that assist the management of cancer data and the interoperability of different hospitals and research centres.

Both caBIG and CancerGrid have developed tools that assist in the development of standardised methodologies for data integration and sample tracking in the context of cancer clinical trials. In each case, there has been an emphasis upon standardising the way in which procedures and observations are described, rather than upon agreeing a single set of common procedures. The only difference is that the process for arriving at standardised descriptions is, at present, centralised in caBIG, and distributed in CancerGrid: in the former, descriptions are standardised by reference to a single, global classification scheme; in the latter, researchers are free to construct a scheme that reflects their local purpose and immediate needs, and then relate this scheme to others as and when necessary. This has meant in turn that there is a greater emphasis in CancerGrid upon support for the evolution of descriptions and protocols - with software artifacts (such as forms and queries) being generated to match successive versions of a model.

In this work, we use the model- or metadata- driven approach employed by the CancerGrid project, since it is best suited for the clinical data integration in the METABRIC project. We do not attempt to transform the data from the five different databases so that they conform to a common description. This would lead to abstracting information out of the data and losing detail that might be important in the interpretation of experiments. Instead, we develop a method that enables us to store the data using their original definitions using Common Data Elements (CDEs).

In order to query the data, we employ SQIV (Crichton, C., *et al In preparation*), a set of CancerGrid tools for Standardization, Querying, Inference and Validation that processes the data using pre-defined inference rules that model the relationships between CDEs.

The problem of integrating data from multiple sources is very similar to those addressed by federated database systems [14,15]; a simplistic view of these is that they allow multiple databases, with different schemas, to be queried using one global schema. In the research context, where different organisations and even individuals may have different views on the relationships between the schemas, this type of system is too restrictive. Instead, we feel that our approach to the classification of data using CDEs, along with mappings between those CDEs, allows us to tackle the issue of semantic heterogeneity whilst giving the individual researcher the freedom to pick the semantics they think is appropriate.

The rest of the paper is structured as follows. First, we provide a description of the Metadata Repository (MDR) implementation and SQIV. Then we describe the model that integrates the five different source database models and we explain how the data is imported and queried. Finally, we discuss the usefulness of the approach and identify directions for future improvements.

## Methods

### Common Data Elements & Metadata Repository

A Common Data Element (CDE) is a metadata definition with an informal explanation of its meaning and usage, a list of alternative names and definitions, units of measurement, and the type of values to be recorded. CDEs can be created for any kind of concept, measurement, or application, and, although grouped into "Data Element Concepts" for convenience, need not derive their meaning from their position in a complex hierarchy or graph. This is in contrast to the ontological approach to data definition, often used in bioinformatics applications [16,17], where each subclass is part of a specification for a representational vocabulary for a particular domain [18]. Although classifications or ontologies can be added to a database of CDEs, they can be used to support navigation and inference on an application-specific basis: there is no requirement to locate a CDE within an existing domain ontology before recording the semantics of a data definition.

The Metadata Repository (MDR) is a database that stores CDEs. The information stored includes the identifier of the CDE and more details such as definition, value domain, unit of measure, property and object class where the CDE belongs to. Its purpose is to address the semantics, the representation and the registration of the descriptions of data. We are using the metadata repository implementation developed by the CancerGrid project http://cancergrid.org/downloads/, whose structure conforms to the ISO/IEC11179 international standard for metadata repositories [19]. The CancerGrid MDR implementation provides tools for registering, updating and browsing CDEs, concepts, properties and their definitions, as well as searching and basic classification tools.

**Figure 1:**
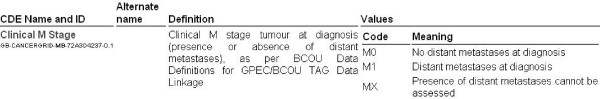
**Clinical M Stage**. An enumerated CDE description of whether there are distant metastases at time of diagnosis.

**Figure 2:**
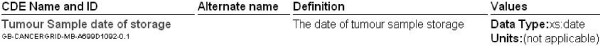
**Tumour Sample Storage Date**. A non-enumerated CDE description of the date when the tumour sample was stored.

**Figure 3:**
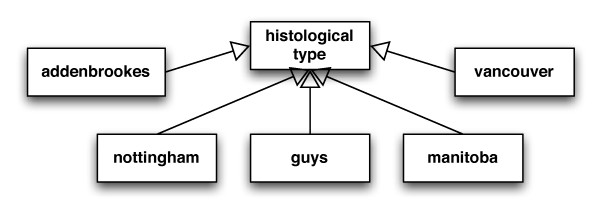
**Histological Type of Tumour**. The histological type of tumour is defined using the different classification systems of the five original databases. 'nottingham' stands for the classification in Nottingham University, 'manitoba' for Manitoba Tumour Bank, 'guys' for Guy's Hospital, 'addenbrookes' for Addenbrookes Hospital, 'vancouver' for Tumour bank of British Columbia (Vancouver).

CDEs either represent enumerated value domains (as in Figure 1) or non-enumerated (as in Figure 2). Every CDE expresses a Data Element Concept. This is a more abstract description of the CDE or several other related CDEs, since a Data Element Concept can be expressed by more than one CDE. An example that illustrates this is the representation of the different 'tumour histological type' classification schemes used by the original databases (Figure 3). Each tumour histological type classification is described by a CDE. The collection of these CDEs express the same Data Element Concept 'histological type'.

### SQIV

In order to compare and query the data in practice, we transform it to some agreed dataset definitions by use of the functions for Data Standardization, Inference and Querying available in SQIV http://cancergrid.org/downloads/sqiv/, a command line java tool.

Standardization is a process that takes data formatted in XML according to an XML schema that is annotated with CDE identifier and produces equivalent RDF for further processing, for example querying or inference. RDF, the Resource Description Framework, is one of the core technologies of the semantic web.

The Querying process takes RDF and allows this to be queried using SPARQL, or to be converted back into XML using an XML schema annotated with CDE identifiers to define the output format.

Finally, the Inference process allows reasoning about semantically annotated data, in order to produce richer data, or data in terms of other meta-data identifiers. In METABRIC, we use the inference function to map the original data definitions to the METABRIC specific ones, as demonstrated in later sections.

## Results

### Clinical Data

The METABRIC project (Molecular Taxonomy of Breast Cancer International Consortium) collects clinical and genomic data on breast cancer tumours from five different hospitals/research centres in the UK and Canada. METABRIC has been approved by the 'NHS National Research Ethics Service, Cambridgeshire 4 Research Ethics Committee' with reference number: 07/MRE05/35. The aim of the project is to generate a robust molecular taxonomy of clinically annotated breast cancers. METABRIC will analyse 2000 breast tumours using a combination of high resolution array-CGH, expression profiling, sequencing and tissue microarray analysis, and correlate the molecular profiles obtained with the clinical outcome of the tumours.

The clinical data used for this study are being obtained from five different sources: in the UK the Cambridge Breast Unit at Addenbrooke's Hospital (Cambridge), Guy's Hospital (London) and Nottingham University City Hospital and in Canada the Tumour Bank of British Columbia (Vancouver) and the Manitoba Tumour Bank. The clinical information required was determined by the aim of the molecular profiling study and it includes survival data, such as date and cause of death, treatment information, such as chemotherapy, surgery and radiotherapy type and bio-marker information such as ER, PR and HER2 status.

Data collection has been a complex process in some cases, since information about a patient was stored in different specialised databases (for example, pathology information, radiotherapy, chemotherapy, clinical trials databases) within the same hospital that were not managed by a central system. The data had to be extracted from all of these and related to each other, in order to achieve a single clinical dataset from each hospital.

Moreover, the vocabularies of the five different data sources differed significantly in parts. There were cases where similar fields were defined using different classification schemes, the most striking example being 'tumour histological type', which was defined differently in each one of the five sources. Other differences included compound measurements that use different types of assessment. For example the Estrogen Receptor levels are recorded using either an immunohistochemistry method or a biochemical assay. Some hospitals record it using one method, others using the other and there are cases where one method was used in earlier breast cancer cases and then it was replaced by a new assessment.

Instead of transforming the data into a common classification scheme for each one of these fields, we create CDEs that record each field definition from each data source. We represent each field of each one of the source databases by a Common Data Element (CDE). This process resulted in 50 CDEs and 33 data element concepts.

In addition to the CDEs that represent each field from each data source, we also record 29 CDEs that define the type of information required by the METABRIC minimum dataset definition (i.e. the clinical information required by the METABRIC study, in the format described by the METABRIC collaborators). CDEs have been curated by the investigators, clinicians and pathologists contributing to the METABRIC study.

### Clinical Database Model

**Figure 4:**
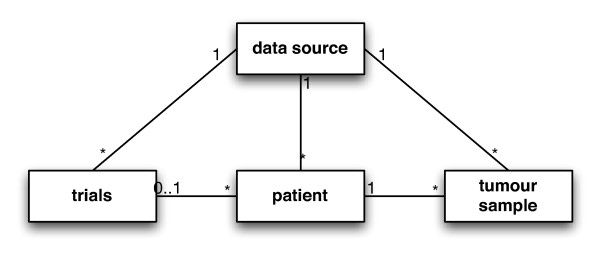
**The Database Model**. The model consists of two main entities, the 'patient' and the 'tumour sample' that contain the clinical and pathological information. Entities 'trials' and 'data source' provide additional information about the trials that patients were part of and the original source of the data.

The database holds the pathological data about the tumour sample and the linked clinical information of the patient. The model of the database contains two main classes 'patient' and 'tumour sample', see Figure 4. Each 'patient' is linked to a 'tumour sample'. A 'patient' can also be linked to 'trials', if appropriate. The class 'data source' holds information about the original databases, from which the data (patient, trial and tumour sample) were extracted. This is the database location information, as well as contact information for the database managers of the original databases.

**Figure 5:**
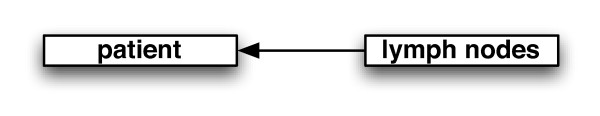
**Compose Relationship**. The complex type element 'patient', contains the complex element 'lymph nodes'. 'lymph nodes' contains the elements 'positive' and 'removed' (integers), corresponding to the number of nodes positive and the number of nodes removed from a patient.

**Figure 6:**
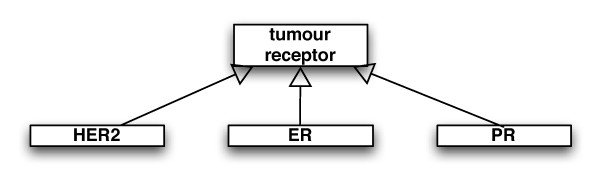
**Generalise Relationship**. The 'HER2', 'PR', 'ER' complex type elements generalise into the 'tumour receptor' element.

The UML model was used to automatically create XML schemas for the different components of the database. The 'patient' XML schema contains all the attributes and XSD Elements within the 'patient' class, as well as all the XSD Complex Type elements linked to the 'patient' XML schema via a 'compose relationship' (Figure 5). This representation allows for 'generalise relationships' between complex type elements. An example is shown in Figure 6, where a 'tumour receptor' can be one of 'HER2', 'PR', 'ER' complex types.

The XML schemas describe the structure of the XML documents that are used to record the information for the patients and tumour samples. Each element on each XML schema is mapped with the appropriate CDE identifier by means of SAWSDL references, making use of the recent extension of XML schema: Semantic Annotations for the Web Services Description Language WSDL (SAWSDL) [20] that supports semantic annotation.

For the reasons discussed earlier in the Background Section, some fields have been defined differently by some or all five sources. For example 'menopausal status at diagnosis' is described as 'clinically determined' in some databases. In others the 'menopausal status at diagnosis' is inferred by the 'age at diagnosis', with patients over 50 years old recorded as post-menopausal and patients less than 50 described as pre-menopausal.

Both 'inferred' and 'clinical' elements are modeled as parts of the complex element 'menopausal status at diagnosis' to record the data according to the format in which it was described by the original database.

However, at the level of XML schema, the 'clinical' menopausal status element is associated with the 'clinical menopausal status' CDE identifier, whereas the 'inferred' menopausal status element is associated with the 'inferred menopausal status' CDE identifier.

Similarly, in the cases of 'histological type of tumour' and 'relapse type', different databases have used different classification schemes. Some databases use the same classification for all three types of relapse (local, regional, distant). Others only record distant relapse type (using a different classification scheme) and others record local and distant relapse types only, again using different classification schemes. The different classification schemes are modeled as sub-classes to a more general class 'relapse', in a similar fashion to the 'menopausal status at diagnosis' element.

### Importing Data

The clinical datasets that are used in METABRIC are received in batches, in tabular format from the different hospital databases. Direct access to the clinical databases is not granted due to the participating hospitals' security regulations involving the protection of patient information. The tabular data sets are then converted into XML documents according to the related CDE-annotated XML schemas in a semi-automated manner, using the built-in functionality of excel or relational database management systems for XML export and XSLT documents. The specifics of the transformation method depend on the schema and data vocabulary of the data source.

Using the inference functionality of SQIV we can pre-process the data in an automatic way, in order to perform any transformations that do not result in information loss but will decrease redundancy in the database (e.g. transform all tumour size values to centimeters). In more detail, we first standardize the data to an RDF format. The Standardization function of SQIV takes as input the XML document containing the original data, the XML schema document that is annotated with the CDE identifiers for each element and outputs an RDF file that contains the mappings between the XML data and the CDE identifiers. Once the data is standardized, we can use the inference tool of SQIV in order to make any transformations required. For this we develop rules that map the CDE annotated data to values of the agreed METABRIC CDEs using the Jena Semantic Web Framework [21]. The Jena Semantic Web Framework is a Java API, employed by the inference tool of SQIV that performs reasoning and in this case transforms the data according to our rules. The result is an RDF file with the new CDE annotations and values, which can then be converted to XML using the Querying tool of SQIV and a METABRIC XML schema. The resulting XML documents are then stored into an eXist database [22].

An example illustrating data transformation that uses SQIV is the mapping of "Nodal Radiotherapy" CDE with values (yes/no) and "Local Radiotherapy" CDE with values (yes/no) from the Nottingham dataset into a combined "Chest Wall-Lymph Node Radiotherapy" CDE created for METABRIC with values (CW (Chest Wall)/Nodal/CW-Nodal/None). Figure 1 and Additional file 1 show the standardized data before inference and Figure 8 shows the data after the inference process.

**Figure 7:**
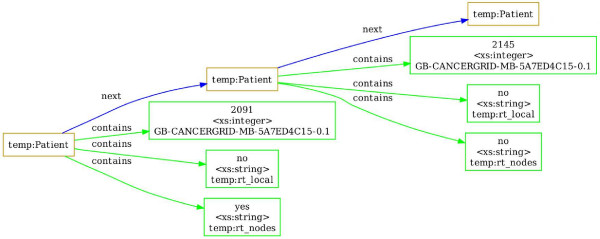
**The structure of standardized data**. The structure of standardized data for two patients including: patient ID (CDE id: *GB-CANCERGRID-MB-5A7ED4C15-0.1*), local radiotherapy (CDE id: *temp:rt_local*) and nodal radiotherapy (CDE id: *temp:rt_nodes*). The original graph on four patients is availbale in Additional File 1. The graph was drawn using GraphViz and the input file to GraphViz was created by using the DOT output format option of SQIV.

**Figure 8:**
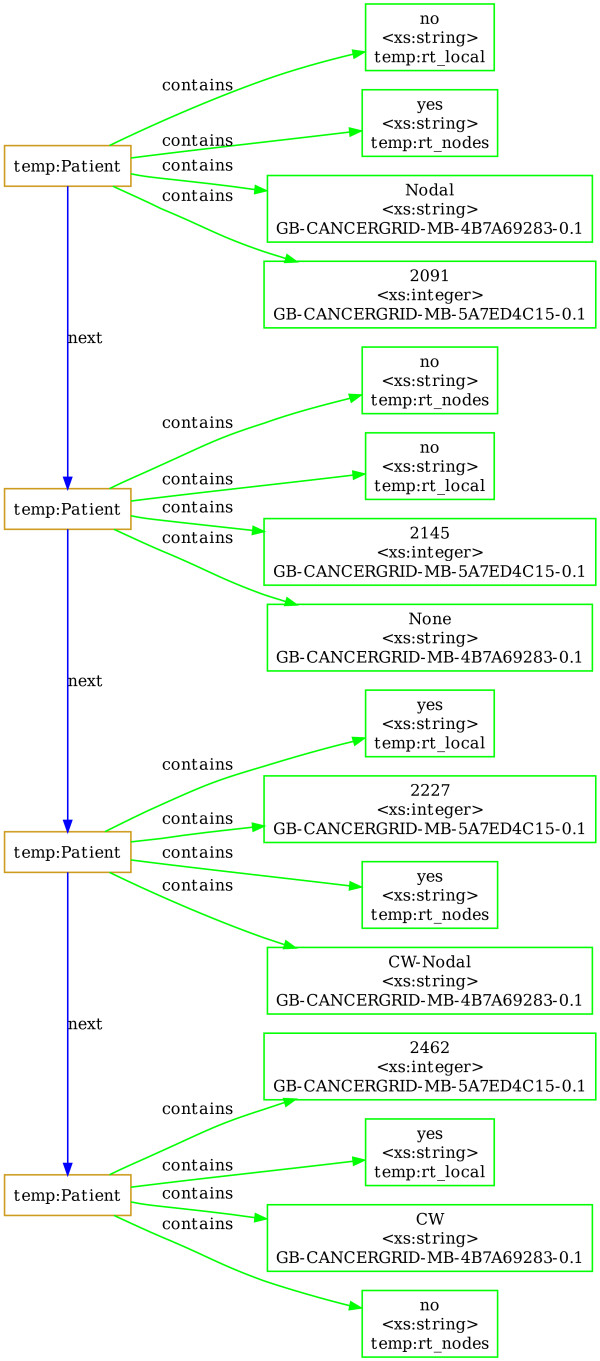
**The structure of inferred data**. The structure of inferred data for four patients including: patient ID (CDE id: *GB-CANCERGRID-MB-5A7ED4C15-0.1*), local radiotherapy (CDE id: *temp:rt_local*), nodal radiotherapy (CDE id: *temp:rt_nodes*) and the inferred CDE Chest wall-Lymph Node Radiotherapy (*GB-CANCERGRID-MB-4B7A69283-0.1*).

### Querying Data

The eXist database system, where the clinical data is stored, supports standard querying tools for XML data, such as XQuery and XPath. However, in order to query across differently defined data fields that are annotated with CDEs we employ the use of reasoning tools.

**Figure 9:**
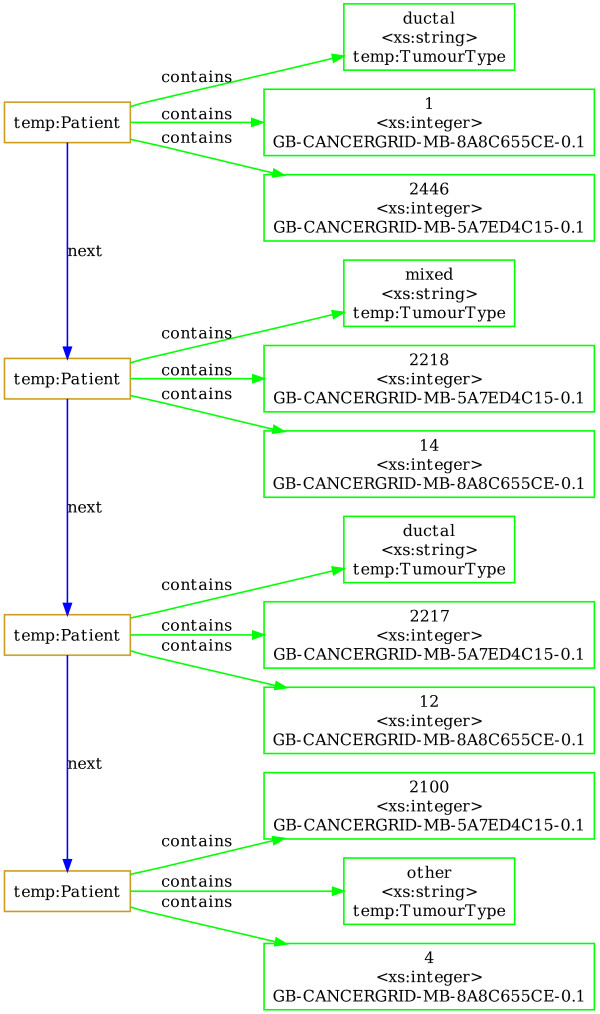
**Inferred data of tumour histological type**. The structure of inferred data for four patients including: patient ID (CDE id: *GB-CANCERGRID-MB-5A7ED4C15-0.1*), Nottingham histological type of tumour (CDE id: *GB-CANCERGRID-MB-8A8C655CE-0.1*) and the inferred CDE 'Tumour Type' (*temp:TumourType*). The meanings of the numerical values of the CDE representing the nottingham histological type of tumour are: 1 for 'Invasive ductal/No special type', 14 for 'Mixed nst and lobular', 4 for 'Atypical medullary' and 12 for 'Invasive cribriform'.

Once the data has been transformed according to a METABRIC schema, we can use SQIV to query across differently defined data fields by mapping the differently defined data fields into a common classification scheme. An example that illustrates how this transformation can take place is the following: The histological type of tumour is a field that is described using classification schemes of different detail across the five data sources in METABRIC. We formulate rules that map the differently defined data fields into a common classification scheme using the Jena Semantic Web Framework. The data is then transformed according to those rules using the inference tool of SQIV. Figure 9 shows an example of four patients whose histological tumour type is recorded according to "Nottingham classification" and how its values (1-15) correspond to the more general "Histological Type of Tumour" CDE with ID *temp:TumourType*. The integers in the Nottingham classification for histological type of tumour correspond to tumour type descriptions and can be found in the data dictionary of the Nottingham Database. Their mappings to more general "Histological Types of Tumour" were made with the assistance of a pathologist.

## Discussion

This work demonstrates how data from different sources can be integrated using semantic annotation, in order to support a large scale collaborative study. For the METABRIC study we developed 79 CDEs that correspond to 33 different data element concepts. Currently, we are testing the method on larger datasets, of about 2000 patients, as we are collecting clinical data from the five collaborating centres of the project and populating the database. At the same time we are enriching the set of inference rules to cater for more cases and we are testing more thoroughly the inference and standardisation functionalities of SQIV. Our experience with the import of 2000 patient records, in batches ranging from 300 to 900 records from five different hospitals has so far been successful.

For the quantities of information we are dealing with in METABRIC (and related research studies) the integration process and the technology used scale up satisfactorily. Integrating more heterogeneous data from a significantly larger number of sources would require the creation of a large number of inference rules that relate CDEs. The speed of SQIV inference would decrease with larger number of records and/or more complex Jena rules. Such cases, dealing with millions of records and a large number of CDE relations, may be tackled by standard optimisation methods and caching.

The ISO/IEC11179 standard for metadata registries employed here provides a simple metadata schema for elements, concepts, value domains and properties. Issues associated with the ISO/IEC11179 include the lack of any structuring of data elements, apart from their association with data element concepts. The question on how to extend (or simplify) ISO/IEC11179 to allow for information structuring in a usable and understandable way is a subject of ongoing research [23]. Here, we were able to overcome this issue by formulating rules in Jena that relate multiple CDEs and deduce the values of inferred CDEs.

The value of this work, as we see it, is that it enables researchers to create and use CDEs and also provide semantics describing the relationship between CDEs that is appropriate to the scope of their current research project. We believe that this 'bottom-up' approach is particularly useful in a research environment, where definitions and semantics change frequently. Different CDEs, as well as their subsequent versions, are given unique identifiers and the relations between them can be formulated on demand and according to their usage, while older versions of CDEs and relations are stored.

Alternative 'top down' approaches, such as ontologies, could be used to model the relationships between CDEs in a more systematic way. Such methods would require that the relationships between CDEs are   defined in advance and could be limiting in research situations, where competing views about what relationships between CDEs must exist.

In this context, and after our experience with the inference rules in METABRIC, the main direction for future work is on structuring the information between CDEs, by storing the relationships between them at a higher level that reflects the inference patterns that are actually being used. We plan to construct an ontology of CDE relations, reflecting the Jena rules used in METABRIC with the view of developing a generalised approach of extracting a structure of CDEs from the existing relations between them. Further planned improvements to the metadata registry and SQIV tool include improving support for XML schema custom simple types and their use for data validation and support for the direct standardization of a wider range of data sources.

## Conclusion

Researchers and clinicians in different hospitals and research centres use different ways of describing and representing their data, for reasons ranging from adhering to a certain local (or national) terminology to the scope of the database they develop. Moreover, these data descriptions change regularly as new methods for medical tests become available. Semantic annotation of the data, ensures that meaning and usage of the data is recorded and enables re-use of the data, and integration with other resources.

We described a general approach for integrating and managing similarities and differences between different datasets using CDEs and inference rules. This approach ensures no information loss, since the datasets are stored according to their original definitions and classification schemes, but at the same time enables data comparison. We apply the approach on clinical data where concepts are described using different classification schemes. However, the approach can be applied for integrating any type of data where similar concepts have been described differently or the data is incomplete.

## Competing interests

The authors declare that they have no competing interests.

## Authors' contributions

IP developed the method with input from LM and PM. ChCr provided expertise on the use of SQIV and also adapted its functionality to the needs of this work. The method was conceived out of discussions between all of the authors. IP wrote the manuscript. All authors read, revised and approved the final manuscript.

## Pre-publication history

The pre-publication history for this paper can be accessed here:

http://www.biomedcentral.com/1755-8794/2/66/prepub

## Supplementary Material

Additional file 1 **The structure of standardized data**. The structure of standardized data for four patients including: patient ID (CDE id: *GB-CANCERGRID-MB-5A7ED4C15-0.1*), local radiotherapy (CDE id: *temp:rt_local*) and nodal radiotherapy (CDE id: *temp:rt_nodes*). The graph was drawn using GraphViz and the input file to GraphViz was created by using the DOT output format option of SQIV. Click here for file
