# Supplementary figures and images for: A metadata approach for clinical data management in translational genomics studies in breast cancer
Source: BMC Med Genomics. 2009 Nov 30;2:66. doi: 10.1186/1755-8794-2-66 (PMC3225860; doi:10.1186/1755-8794-2-66)

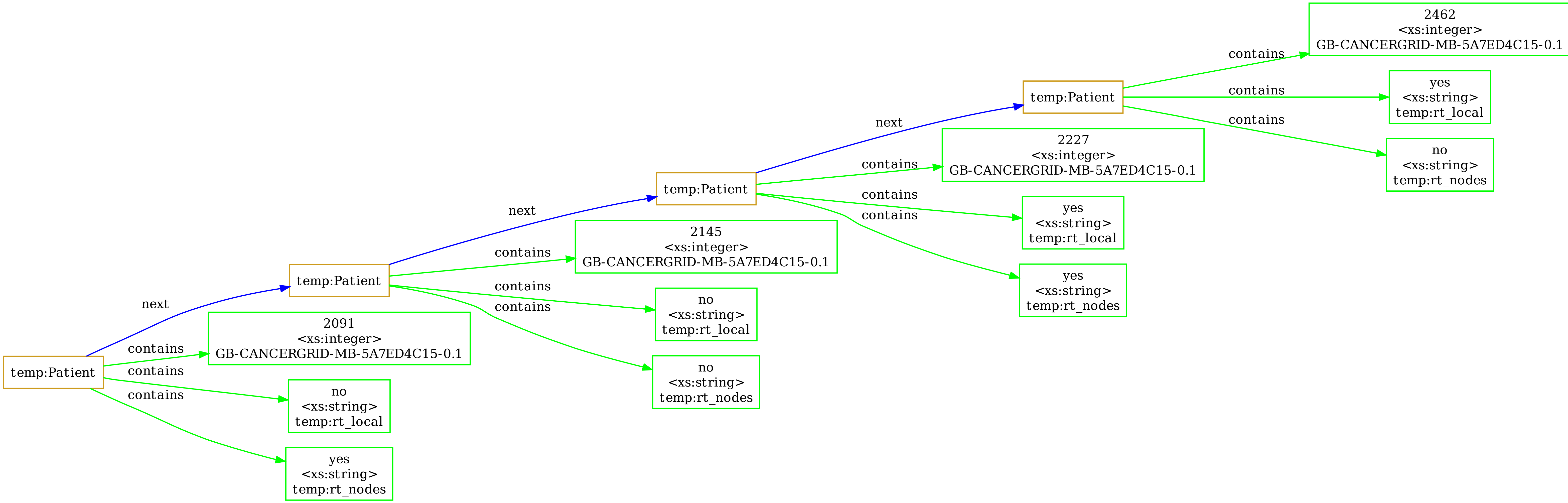

Supplement: Additional file 1 — The structure of standardized data. The structure of standardized data for four patients including: patient ID (CDE id: GB-CANCERGRID-MB-5A7ED4C15-0.1), local radiotherapy (CDE id: temp:rt_local) and nodal radiotherapy (CDE id: temp:rt_nodes). The graph was drawn using GraphViz and the input file to GraphViz was created by using the DOT output format option of SQIV. [file 1755-8794-2-66-S1.PDF]
